# Outcomes After Surgical Resection of Primary Non-Myxoma Cardiac Tumors

**DOI:** 10.21470/1678-9741-2017-0152

**Published:** 2018

**Authors:** Kamil Boyacıoğlu, Adnan Ak, Arzu Antal Dönmez, Burçin Çayhan, Mehmet Aksüt, Mehmet Altuğ Tunçer

**Affiliations:** 1 Cardiovascular Surgery Department, Bagcilar Research and Training Hospital, Istanbul, Turkey.; 2 Kartal Koşuyolu Research and Training Hospital, Cardiovascular Surgery Department, Istanbul, Turkey.

**Keywords:** Heart Neoplasm, Cardiovascular Surgical Procedures, Treatment Outcome

## Abstract

**Objective:**

Primary cardiac tumors are rare lesions with different histological type. We reviewed our 17 years of experience in the surgical treatment and clinical results of primary non-myxoma cardiac tumors.

**Methods:**

Between July 2000 and February 2017, 21 patients with primary cardiac tumor were surgically treated in our institution. The tumors were categorized as benign non-myxomas and malignants. Data including the demographic characteristics, details of the tumor histology and grading, cardiac medical and surgical history, surgical procedure of the patients were obtained from the hospital database.

**Results:**

Eleven patients were diagnosed with benign non-myxoma tumor (male/female:7/4), ranging in age from 10 days to 74 years (mean age 30.9±26.5 years). Papillary ﬁbroelastoma was the most frequent type (63.6%). There were two early deaths in benign group (all were rhabdomyoma), and mortality rate was 18%. The mean follow-up period was 69.3±58.7 months (range, 3 to 178 months). All survivals in benign group were free of tumor-related symptoms and tumor relapses. Ten patients were diagnosed with malignant tumor (sarcoma/lymphoma:8/2, male/female:3/7), ranging in age from 14 years to 73 years (mean age 44.7±18.9 years). Total resection could be done in only three (30%) patients. The mean follow-up period was 18.7±24.8 months (range, 0-78 months). Six patients died in the first 10 months.

**Conclusion:**

Complete resection of the cardiac tumors, whenever possible, is the main goal of surgery. Surgical resection of benign cardiac tumors is safe, usually curative and provides excellent long-term prognosis. On the contrary, malignant cardiac tumors still remain highly lethal.

**Table:** 

Abbreviations, acronyms & symbols	
TTE	= Transthoracic echocardiogram

## INTRODUCTION

Cardiac tumors can be categorized as primary or secondary, depending on the origins of the tumors. Metastatic malignant heart tumors are nearly 50 times more common than primary cardiac tumors^[1]^. The presence of primary cardiac tumors is rare with an overall incidence rate of < 0.33%. Approximately 75% of the primary cardiac tumors are benign; the most common histopathological type is myxoma in 50% of the cases, followed by papillary fibroelastomas, fibromas, lipomas, rhabdomyomas, hemangiomas and teratomas^[2]^. Cardiac sarcoma represents the primary malignant tumors of the heart. Lymphoma can also affect the heart, primarily^[3]^. The symptoms of the tumors are non specific and can mimic many other cardiac diseases.

In this study, we reviewed our 17 years of experience in the surgical treatment and clinical results of primary non-myxoma cardiac tumors.

## METHODS

Between July 2000 and February 2017, 21 patients with primary cardiac tumors were surgically treated in our institution and 11 of these were diagnosed with primary benign cardiac tumors. Patients with tumors metastatic to the heart and those in whom a primary cardiac origin was not clear were excluded from the study. Data including the demographic characteristics, details of the tumor histology and grading, cardiac medical and surgical history, surgical procedure of the patients were obtained from the hospital database. Preoperative diagnosis was established using transthoracic echocardiogram (TTE). Coronary angiography and computed tomography were performed in eight (38%) and three (14%) patients, respectively. Annual TTEs were performed during follow-up to evaluate the left ventricular function, presence of intracardiac masses, filling status and valve function after tumor resection. Patients were interviewed over the phone to assess late functional status. Statistical analyses were performed using the statistical software SPSS 15.0 for Windows (SPSS Inc, Chicago, IL, USA). Pearson chi-square test was used for comparisons between the pediatric and adult patients. Survival was estimated using the Kaplan-Meier product limit method and curves were compared using a log-rank test. A *P*-value <0.05 was considered statistically significant.

For reporting purposes, the tumors were grouped into two categories: benign non-myxomas and malignant.

The study protocol was approved by the Kartal Koşuyolu Research and Training Hospital Ethics Committee. Written informed consents were obtained from each patient or parents, in case of pediatric patients. The study was conducted in accordance with the principles of the Declaration of Helsinki. The authors had full access to all of the data in this study and take full responsibility for the integrity of the data. All authors have read and agreed with the manuscript.

## RESULTS

Benign non-myxoma tumors represent 52% of the total patient cohort, seven (64%) patients were male and four (36%) were female, ranging in age from 10 days to 74 years (mean age 30.9±26.5 years). Seven patients had papillary ﬁbroelastomas and other pathology as delineated in Table 1. There was a female predominance (female/male:7/3) in patients with malignant tumors and they were ranging in age from 14 years to 73 years (mean age 44.7±18.9 years). Two patients had primary cardiac lymphomas and rest had different kinds of sarcomas (Table 1).

**Table 1:** Histopathologic types of tumors.

Histology	Number of patients
Benign	
Papillary fibroelastoma	7
Rhabdomyoma	2
Fibroma	1
Angiomatosis	1
Malignant	
Angiosarcoma	2
Leomyosarcoma	2
Rhabdomyosarcoma	2
Fibrosarcoma	1
Sarcoma	1
T cell lymphoma	1
B cell lymphoma	1
Total	21

The symptoms, surgical procedures and follow-up periods of all patients in both groups are summarized in Tables 2 and 3. Dyspnea and palpitation were the most frequent symptoms. Although some patients with papillary fibroelastoma were asymptomatic in benign group, there were various grades of symptoms in all of the malignant types.

**Table 2:** Clinical findings, surgical procedures and follow-up of benign non-myxoma cardiac tumors.

	Age/Gender	Symptoms	Pathology/Size	Location	Approach	Surgery	Survey/Follow-up
1	28 years, M	Angina	Papillary fibroelastoma/1x1 cm	AV	Aortotomy	Resection	Survived/3 months
2	74 years, M	Asymptomatic	Papillary fibroelastoma/1x1.2 cm	AV	Aortotomy	Resection, aortic repair	Survived/3 months
3	28 years, M	Palpitation	Papillary fibroelastoma/1x1.5cm	MV posterior leaflet	Right atriotomy, transseptal left atriotomy	Resection	Survived/35 months
4	67 years, M	Dyspnea, angina	Papillary fibroelastoma/1.5x1.5 cm	TV posterior leaflet	Right atriotomy	Resection, tricuspid repair, CABG	Survived/38 months
5	9 years, F	Asymptomatic	Papillary fibroelastoma/1x1 cm	MV papillary muscle	Left and right atriotomy	Resection, PFO repair	Survived/51 months
6	52 years, F	Dyspnea	Papillary fibroelastoma/1x1 cm	MV papillary muscle	Left atriotomy	Resection, mitral ring annuloplasty	Survived/119 months
7	51 years, F	Asymptomatic	Papillary fibroelastoma/1x1 cm	AV	Aortotomy	Resection, aortic repair	Survived/178 months
8	1 month, M	Dyspnea	Rhabdomyoma/4x4 cm	RVOT	Right atriotomy and right ventriculotomy	Complete resection, RV patch repair	Died /postoperative day 1
9	10 days, M	Dyspnea, cyanosis	Rhabdomyoma/4x5 cm	LV	__	İncomplete resection	Died/2 months
10	5 years, F	Dyspnea, palpitation	Fibroma/5.5x3 cm	LV	__	Complete resection	Survived/112 months
11	25 years, M	Palpitation, syncope	Angiomatosis/5x5 cm	LV	Left ventriculotomy	Complete resection	Survived/85 months

AV=aortic valve; CABG=coronary artery bypass surgery; F=female; LV=left ventricle; M=male; MV=mitral valve; PFO=patent foramen ovale; RVOT=right ventricle outflow tract; TV=tricuspid valve

**Table 3:** Clinical findings, surgical procedures and follow-up of malignant cardiac tumors.

	Age/ Gender	Symptoms	Pathology/Size	Location	Approach	Surgery	Survey/Follow-up
1	43 years, F	Dyspnea, palpitation	Rhabdomyosarcoma/ 6x5 cm	LA	Left and right atriotomy	Incomplete resection	Died/9 months
2	73 years, F	Palpitation,dsypnea, TİA	Rhabdomyosarcoma/ 2x2 cm	LA	Left and right atriotomy	Complete resection, mitral ring annuloplasty	Died/3 months
3	30 years, F	Dyspnea	Leomyosarcoma/ 3x5 cm	RA, RV, RVOT	Right atriotomy	Incomplete resection (reoperation at 30^th^ and 39^th^ months)	Died/43 months
4	49 years, F	Dyspnea, palpitation	Leomyosarcoma/ 7x8 cm	LA	Left atriotomy	Complete resection, mitral valve repair	Died/ 28 months
5	23 years, M	Dyspnea, palpitation, syncope	Angiosarcoma/ 8x9 cm	RA, RV	Right atriotomy	Incomplete resection	Died/5 months
6	47 years, F	Dyspnea, syncope	Angiosarcoma/ 10x4 cm	RA	Right atriotomy	Complete resection, TDVA, CABG	Survived/10 months-NED
7	62 years, F	Dyspnea, palpitation, peripheral edema	Fibrosarcoma/9x6.5 cm	RV, PA	Right ventriculotomy, pulmonary arteriotomy	Incomplete resection, pulmonary endarterectomy	Died/ postoperative day 5
8	40 years, F	Dyspnea, palpitation, peripheral edema	Sarcoma/7x3 cm	LA, LV	Left and right atriotomy, aortotomy	Incomplete resection	Died/10 months
9	14 years, M	Dyspnea, palpitation	T cell lymphoma/ 6x8 cm	SVC, aorta, pulmonary artery	___	Incomplete resection	Survived/78 months-NED
10	66 years, M	Dyspnea	B cell lymphoma/ 9x9.5 cm	RA, RV	Right atriotomy	Incomplete resection	Died/1 month

CABG=coronary artery bypass surgery; F=female; LA=left atrium; LV=left ventricle; M=male; NED=no evidence of disease; PA=pulmonary artery; RA=right atrium; RV=right ventricle; RVOT=right ventricle outflow tract; SVC=superior vena cava; TDVA=tricuspid De Vega annuloplasty

There were five pediatric patients in the entire group and four of these patients had benign tumors (Table 4). As seen on Table 4, although malignant primary cardiac tumors appeared almost exclusively in adults in our patients, no statistically significant difference between the pediatric and adult patients could be observed (*P*=0.157). The details of tumor types in pediatric patients are shown in Tables 2 and 3.

**Table 4:** The distribution of the tumor types according to age groups.

	Benign tumors (n=11)	Malignant tumors (n=10)	*P*
Pediatric patients	4	1	0.157
Adult patients	7	9	

Three patients suffered from mitral valve regurgitation, one patient had severe anterior descending artery stenosis, right ventricular outﬂow tract obstruction occurred in one patient, and patent foramen ovale was present in one patient. Two patients had previous extracardiac malignancy: gastric lymphoma and fibrosarcoma in abdomen (patient 1 in malignant, patient 4 in benign group, respectively).

Resection of the cardiac tumors was performed through a median sternotomy with cardiopulmonary bypass in all patients except three: patient 9 in benign, and patients 5 and 9 in malignant group. In all three patients palliative resection was performed as much as possible and chemotherapy was administered after surgery. Ascending aortic arterial and bicaval venous cannulation was the standard technique with moderate systemic hypothermia (30º-32ºC). In patients with aortic papillary fibroelastoma two-stage venous cannulation was done and in one patient with fibrosarcoma total circulatory arrest was required for the resection of the tumor invading pulmonary artery branches. Both pediatric patients with rhabdomyoma underwent emergency surgery because of the right ventricular outflow tract obstruction (patient 8) and congestive heart failure with severe cyanosis (patient 9). The latter patient had hypoplasia of the left lung and only palliative resection could be done without the use of cardiopulmonary bypass. In one patient in malignant group with unclassified sarcoma diagnosis (patient 8), emergent surgery was required due to the presence of pulmonary edema. With the rest of the patients, operations were undertaken on an urgent basis (within one week). In benign group, entire tumor mass could be resected completely except for one (91%) patient, but in malignant group total resection could be done in only three (30%) patients. Associated procedures: three mitral valve annuloplasties, two tricuspid valve annuloplasties (De Vega and bicuspidization technique), two coronary artery bypass grafts (due to the complete resection of angiosarcoma that has spread around the right coronary artery route in patient 6), two aortic valve repairs and one patent foramen ovale repair.

Mean cardiopulmonary bypass time was 73.4±55.1 min and mean myocardial ischemic time was 35.2±11 min in benign group. Those times were 107.6±55 min and 74.3±44.6 min in malignant group, respectively. Mean duration of hospital stay was 6.5±1.5 days and mean mechanical ventilation time was 5.7±2.8 h in benign group and these times were 10.2±4.9 days and 11±6.2 h. in malignant group, respectively.

Arrhythmia was observed in two patients in malignant group (patient 4 and 8) after surgery. One patient suffered from pneumonia in postoperative period (patient 2 in malignant group). Postoperative bleeding occurred in three patients. Drainage was successfully managed medically in the patient with T-cell lymphoma, but the other two were needed re-exploration (patient 2 in benign, patient 7 in malignant group). Extracorporeal membrane oxygenation and high-dose inotropic support was required in the postoperative period due to low-cardiac output after the resection of a large, right ventricular and pulmonary arterial fibrosarcoma in one patient (patient 7).

Two of the deceased patients had benign tumors (all were rhabdomyoma) and eight had malignant tumors with postoperative mortality rates of 18% and 80%, respectively.

**Fig. 1:**
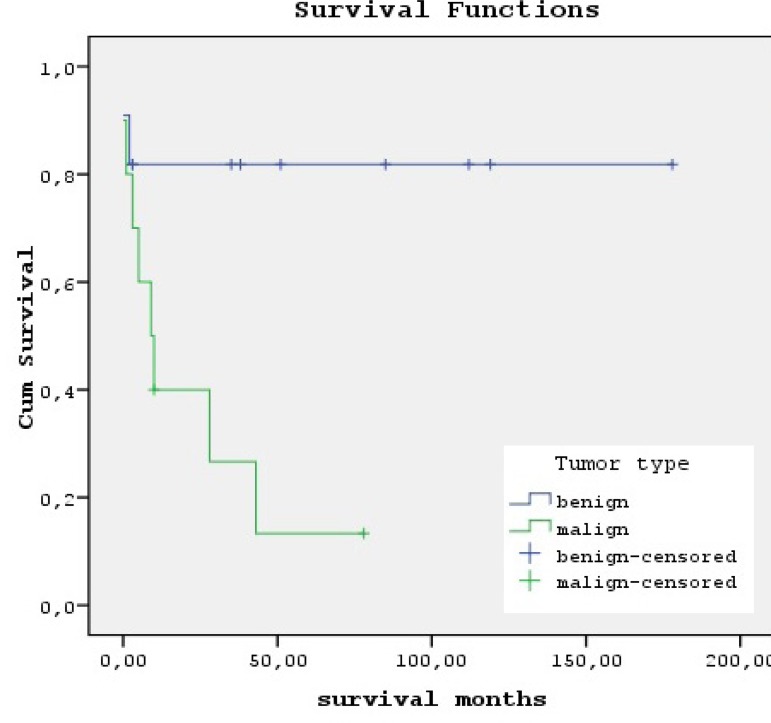
Survival curves of patients with benign non-myxoma (blue) versus malign cardiac tumors (green) demonstrate an inferior survival for patients with malign tumors (P=0.017).

**Fig. 2:**
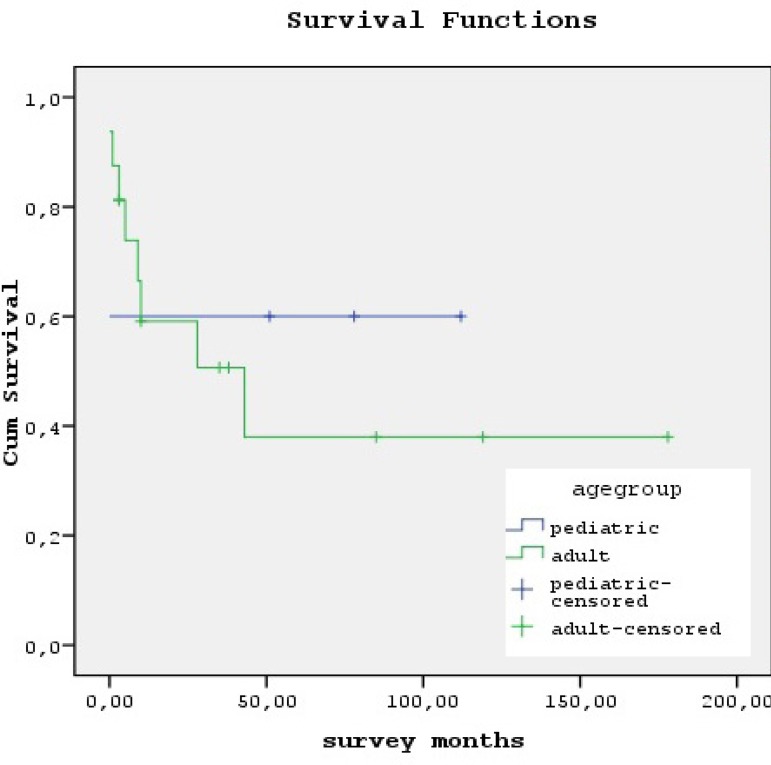
Survival curves between the pediatric (blue) and adult (green) patients. No statistically significant difference between the groups was observed (P=0.717).

All survivals in benign group were free of tumor-related symptoms and tumor relapses or any progression. No patient in this group required reoperation for tumor related or any other cardiac problem. The mean follow-up period was 69.3±58.7 months (range, 3 to 178 months). Two patients with sarcoma had evidence of metastatic disease after surgery (patient 4- lung, patient 7- liver). One patient required reoperation in the 30^th^ and 39^th^ months during the follow-up period (Table 3). The patient with T-cell lymphoma had complete remission after the chemotherapy. He is now 20 years old with no evidence of lymphoma in any screening modalities. The other survived patient with angiosarcoma has no tumor-related clinical manifestation. She is receiving chemotheraphy with anthracycline and ifosfamide. The mean follow-up period for the entire group was 18.7±24.8 months (range, 0-78 months). Six patients died in the first 10 months. The cumulative survival rates are shown in Figure 1 and this finding was statistically significant (*P*=0.017 by log-rank test). In pediatric patients, there were two early deaths, one patient were diagnosed with rhabdomyoma and the other three patients are still alive without any complaints associated with the tumors. In Figure 2, the cumulative survival rates between the pediatric and adult patients are shown and no statistically significant difference was observed (*P*=0.717 by log-rank test).

## DISCUSSION

Despite the widespread use of echocardiography, primary cardiac tumors remain an uncommon occurrence. Myxomas are conﬁrmed to be the most frequent cardiac tumors and once they are excluded, the other types of cardiac tumors are extremely rare. After surgical resection, the main difference between benign and malignant tumors is the survival rates^[2,4]^. In our series, surgical resection of primary benign cardiac tumors has good short and long term results; conversely the prognosis of malignant types is poor.

Rhabdomyomas are the most frequent primary cardiac tumor in infants and children and are usually discovered in patients less than one year of age^[5]^. These tumors are benign myocardial hamartomas which usually display no significant symptoms and tends to regress spontaneously. Thus, most patients without any hemodynamic abnormalities or intractable arrhythmias do not require routine surgery. Moreover, total resection of the tumor may not be always possible due to the pathologic characteristic of rhabdomyomas: they are intramural masses and most commonly multiple in number with intracavitary extensions^[6]^. In our patients, both had severe hemodynamic instability and total resection could be performed in only one patient, but both died after surgery in the early period. Although the number of patients in the different studies is not sufficient, mortality rates vary between 3-21%^[5,7]^.

Papillary fibroelastomas are the most frequent cardiac valvular tumor. Although there are many hypotheses, the etiology is still unclear^[8]^. They are usually solitary, less than 1.5 cm in diameter, and diagnosed in older patients. As with our patients, sometimes they can be encountered in children and young adults^[7,9]^. There is no gender predominance. Most papillary fibroelastomas are asymptomatic and found incidentally, however, affected patients may present with thromboembolic events such as transient ischemic attacks or stroke, chest pain or sudden death secondary to the obstruction of coronary arteries and peripheral embolism^[8,10]^. These tumor related embolic events and death are associated with mobility of the tumor not with its size^[11]^. The surgical resection is curative and long-term prognosis after surgery is excellent^[2,11]^.

Cardiac fibromas typically occur in childhood period. These tumors are solitary and usually arise from left ventricle as in our case. They may invade the ventricle muscles and the conduction system, therefore they can cause the congestive heart failure and refractory ventricular arrhythmias. Surgery is usually indicated because spontaneous regression has never been observed and surgical outcome is successful in most of the patients whether total or partial resection has been done with freedom from recurrence^[12]^.

Hemangiomas and lymphangiomas are major vascular cardiac tumors. Angiomatosis is quite a rare vascular tumor and is usually observed during childhood. Primary left ventricular angiomatosis was first described in our patient^[13]^. They may cause ventricular tachycardia and presyncopal attacks. The tumor was capsulated, yellowish and rubbery in our case. During the follow-up period, no recurrence or relapse or any other hemodynamic concerns were encountered.

Approximately 10% of cardiac tumors which are surgically resected are primary sarcomas. Sarcomas are malignant mesenchymal tumors. Angiosarcomas are the most common type of primary cardiac sarcomas^[14]^. Although they can involve all of the cardiac chambers, angiosarcomas tend to arise from the right atrium, the other types of sarcomas more commonly affect the left side of the heart. Patients can present with similar symptoms as the other cardiovascular diseases: dyspnea (the most frequent symptom), palpitation, chest pain, congestive heart failure, pericardial effusion or cardiac tamponade. Today, after the diagnosis of cardiac sarcoma to be established, patients undergo a thorough review by a multidisciplinary cardiac tumor team, including medical and radiation oncologists, cardiologists, radiologists and cardiac surgeons. Multiple imaging modalities including echocardiography, computed tomography, cardiac magnetic resonance imaging, total body positron emission tomography, coronary angiography are utilized to assess the cardiac structures, tumor resectability and intra- or extrathoracic metastases and to plan the treatment^[15,16]^. Treatment consists of neoadjuvant chemotherapy, radical surgery and adjuvant chemo- and radiation therapy. Primary cardiac sarcomas possess the highly aggressive local growth and metastatic spreads are common. The prognosis for patients with cardiac sarcomas remains very poor. Survival rates are primarily related with tumor histology, complete or incomplete resection and distant organ metastasis at presentation^[14,17]^. Some authors classified the cardiac sarcoma by their anatomic location rather than by histopathologic type: right heart sarcomas, left heart sarcomas, pulmonary artery sarcomas^[15,16,18]^. Right heart sarcomas tend to be bulky, infiltrative and metastasize early and neoadjuvant chemotherapy can reduce the tumor size thus increase the possibility of success a total resection; in contrast, left heart sarcomas typically presenting with locally advanced tumor and cardiac autotransplantation technique may enable the optimal exposure for complete resection and cardiac reconstruction in complex left sided tumors^[18,19]^. Pulmonary artery sarcoma tends to be angiosarcoma and present with symptoms related to pulmonary artery obstruction associated with intraluminal growth and spread. This classification influence the prognosis and treatment strategy rather than by histological type^[15,16,20]^. Furthermore, neoadjuvant and/or adjuvant chemotherapies have been reported to achieve prolonged survival rates in certain cases^[15,16,20]^.

Primary cardiac lymphoma is an extremely rare subset of non-Hodgkin's lymphoma, involving only the heart and/or the pericardium. The majority of cardiac lymphomas are B-cell neoplasms^[21]^. T-cell lymphoma was found in only 5% of all primary cardiac lymphoma types in a large literature review^[22]^. These tumors generally involve the right heart and the right atrium is the cardiac chamber most affected by primary cardiac lymphoma^[3]^. Lymphoma may appear isolated in the mitral valve^[23]^. In our cases, one of them arose from the right atrium and ventricle, and the other originated from the superior vena cava. The latter has spread to the aorta and pulmonary artery. Dyspnea is the most common presenting symptom in the primary cardiac sarcomas. The most common treatment modality is chemotherapy and the surgical resection is required almost 30% of patients^[22]^. The prognosis of primary cardiac lymphomas is better than the cardiac sarcomas in treated patients and surgery accompanied by chemotherapy has good outcomes^[24,25]^. Immunodeficiency, extracardiac disease, left ventricular involvement, and the absence of arrhythmias have adverse effect on prognosis^[22]^.

### Limitation

The findings of a retrospective study of patients that underwent surgical resection of primary non-myxoma cardiac tumors were presented. The major limitation of this study was its small sample size in both benign and malignant groups because of the rarity of these tumors. Moreover, we could only perform palliative surgery in most of the patients in the malignant group and so the prognostic significance between complete and incomplete resection was not evaluated in the follow-up period. Over the years, many different surgeons have participated in these operations in our department. Although these tumors are rare; after the diagnosis, especially in patients with cardiac sarcomas, the realization of these operation performing of these operations by experienced surgeons in a multidisciplinary approach may improve patient outcomes.

## CONCLUSION

Primary cardiac tumors have many different histopathological types in benign and malignant groups. Complete resection of the tumor, whenever possible, is the main goal of surgery. Surgical resection of benign cardiac tumors is safe, usually curative and provides excellent long-term prognosis. On the contrary, malignant cardiac tumors still remain highly lethal. Adjuvant therapy may increase survival rates in primary cardiac lymphoma.

**Table:** 

Authors' roles & responsibilities	
KB	Substantial contributions to the conception or design of the work; or the acquisition, analysis, or interpretation of data for the work; final approval of the version to be published
AA	Substantial contributions to the conception or design of the work; or the acquisition, analysis, or interpretation of data for the work; final approval of the version to be published
AAD	Substantial contributions to the conception or design of the work; or the acquisition, analysis, or interpretation of data for the work; final approval of the version to be published
BÇ	Substantial contributions to the conception or design of the work; or the acquisition, analysis, or interpretation of data for the work; final approval of the version to be published
MA	Substantial contributions to the conception or design of the work; or the acquisition, analysis, or interpretation of data for the work; final approval of the version to be published
MAT	Substantial contributions to the conception or design of the work; or the acquisition, analysis, or interpretation of data for the work; final approval of the version to be published
